# Effect of antibiotic therapy on the prognosis of ventilator-associated pneumonia caused by *Stenotrophomonas maltophilia*

**DOI:** 10.1186/s13613-021-00950-1

**Published:** 2021-11-26

**Authors:** Bérénice Puech, Clémence Canivet, Laura Teysseyre, Guillaume Miltgen, Thomas Aujoulat, Margot Caron, Chloé Combe, Julien Jabot, Olivier Martinet, Jerome Allyn, Cyril Ferdynus, Nicolas Allou

**Affiliations:** 1 Réanimation Polyvalente, Hôpital Universitaire Félix Guyon, Allée des Topazes, 97400 Saint Denis, France; 2 Service de Microbiologie, Hôpital Universitaire Félix Guyon, Allée des Topazes, 97400 Saint Denis, France; 3 UMR Processus Infectieux en Milieu Insulaire Tropical (PIMIT), CNRS 9192, INSERM U1187, IRD 249, Université de La Réunion, Saint-Denis, France; 4 Département d’Informatique Clinique, Hôpital Universitaire Félix Guyon, Allée des Topazes, 97400 Saint Denis, France

**Keywords:** Ventilator-associated pneumonia, *Stenotrophomonas maltophilia*, Outcome, Antibiotic therapy

## Abstract

**Background:**

Ventilator-associated pneumonia (VAP) caused by *Stenotrophomonas maltophilia* is poorly described in the literature. However, it has been shown to be associated with increased morbidity and mortality. Probabilistic antibiotic therapy against *S. maltophilia* is often ineffective as this pathogen is resistant to many antibiotics. There is no consensus at present on the best therapeutic strategy to adopt (class of antibiotics, antibiotic combination, dosage, treatment duration). The aim of this study was to evaluate the effect of antibiotic therapy strategy on the prognosis of patients with VAP caused by *S. maltophilia.*

**Results:**

This retrospective study evaluated all consecutive patients who developed VAP caused by *S. maltophilia* between 2010 and 2018 while hospitalized in the intensive care unit (ICU) of a French university hospital in Reunion Island, in the Indian Ocean region. A total of 130 patients with a median Simplified Acute Physiology Score II of 58 [43–73] had VAP caused by *S. maltophilia* after a median duration of mechanical ventilation of 12 [5–18] days. Ventilator-associated pneumonia was polymicrobial in 44.6% of cases, and ICU mortality was 50.0%. After multivariate Cox regression analysis, the factors associated with increased ICU mortality were older age (hazard ratio (HR): 1.03; 95% CI 1.01–1.04, *p* = 0.001) and high Sequential Organ Failure Assessment score on the day of VAP onset (HR: 1.08; 95% CI 1.03–1.14, *p* = 0.002).

Appropriate antibiotic therapy*,* and in particular trimethoprim–sulfamethoxazole, was associated with decreased ICU mortality (HR: 0.42; 95% CI 0.24–0.74, *p* = 0.003) and decreased hospital mortality (HR: 0.47; 95% CI 0.28–0.79, *p* = 0.04).

Time to start of appropriate antibiotic therapy, combination therapy, and duration of appropriate antibiotic therapy had no effect on ICU mortality (*p* > 0.5).

**Conclusion:**

In our study, appropriate antibiotic therapy, and in particular trimethoprim–sulfamethoxazole, was associated with decreased ICU and hospital mortality in patients with VAP caused by *S. maltophilia*.

## Introduction

Ventilator-associated pneumonia (VAP) caused by *Stenotrophomonas maltophilia* is relatively common, with the latest report of the European Centre for Disease Prevention and Control stating that this microorganism is one of the 10 most frequently isolated germs in respiratory samples [1]. Depending on the study, the incidence *S. maltophilia* in patients with VAP varies between from 0.3 and 2.0% [2, 3]. According to some authors, however, *S. maltophilia* is of limited pathogenicity and should not be considered as an infectious agent in the majority of VAP cases [4–6]. Admittedly, the link between colonization by *S. maltophilia* and respiratory infection is often difficult to establish. On the one hand, severely ill patients can be colonized with *S. maltophilia* in the respiratory tract without presenting respiratory symptoms [4, 5]. On the other hand, half of respiratory samples containing strains of *S. maltophilia* show significant growth of other microorganisms with known pathogenicity [2, 7]. Several studies nonetheless suggest that VAP caused by *S. maltophilia* is associated with high morbidity and mortality [3, 4, 7], in particular due to inappropriate probabilistic treatment [7, 8]. In some studies, the mortality rate attributable to *S. maltophilia* colonization or infection was found to vary between 20.0 and 38.0% [8].

There is no consensus at present on the therapeutic strategy to adopt in cases of VAP caused by *S. maltophilia* (class of antibiotics, antibiotic combination, dosage, treatment duration). Some authors recommend high doses of trimethoprim–sulfamethoxazole based on clinical data, while others favor combination therapy based on in vitro data [9, 10]. In view of this, our study aimed to evaluate the effect of antibiotic therapy strategy on the prognosis of patients with VAP caused by *S. maltophilia.*

## Methods

This single-center retrospective study was conducted in the Multi-Purpose Intensive Care Unit (ICU) of Félix Guyon University Hospital in Reunion Island, a French overseas department located in the Indian Ocean.

**Fig. 1:**
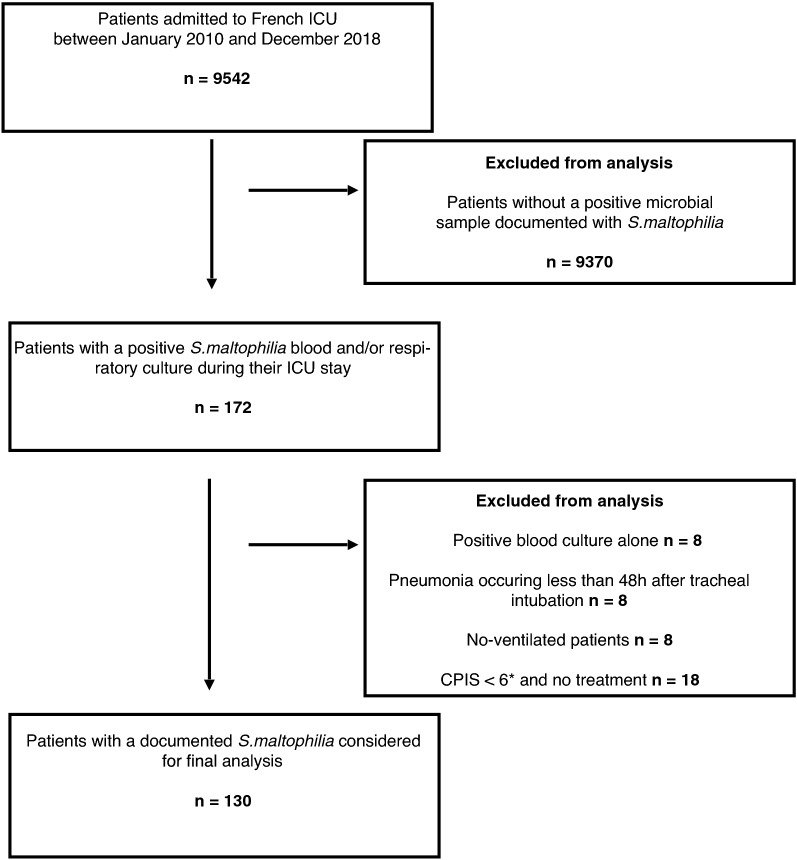
Flowchart

All patients hospitalized in ICU with a blood or respiratory culture positive for *S. maltophilia* between 1 January 2010 and 31 December 2018 were evaluated and considered for inclusion. Patients aged over 18 years who developed VAP caused by *S. maltophilia* during their ICU stay were included in the study (Fig. 1).

In accordance with the French legislation on non-interventional studies [11], this study was registered with the National Institute of Health Data under the number MR 5611200420 and was approved by the Ethics Committee of the French Society of Infectious Disease and Tropical Medicine (CER-MIT 2021-0302). This study complies with the Strengthening the Reporting of Observational studies in Epidemiology recommendations statement [12].

### Definitions

Ventilator-associated pneumonia caused by *S. maltophilia* was defined by: new or progressive infiltrate; temperature > 38.0 °C or < 36.5 °C; white blood cell count > 12G/L or < 4G/L; purulent secretions; drop in oxygenation; positive respiratory culture obtained by bronchoalveolar lavage (threshold: 10^4^ colony forming unit (CFU)/mL), protected distal sampling (threshold: 10^3^ CFU/mL) or endotracheal aspiration (threshold: 10^6^ CFU/mL); and occurrence of infection 48 h or more after tracheal intubation. [13].

All patients with a Clinical Pulmonary Infection Score (CPIS) > 6 and those with a CPIS ≤ 6 who were treated by a clinician for VAP were evaluated [14].

### Pharmacological management

In accordance with our protocol, all patients with VAP caused by *S. maltophilia* were treated with: trimethoprim–sulfamethoxazole (1200 mg/240 mg)/6 h and/or ciprofloxacin 400 mg/8 h and/or moxifloxacin and/or ticarcillin–clavulanate 4 g/8 h and/or ceftazidime 2 g/6 h.

Dosages were adjusted to renal function if necessary, and the choice of antibiotics was left to the discretion of the clinician.

### Data collection

The following information was collected:

- Demographic data (age, sex), organ failure during ICU stay and on the day of VAP onset, and comorbidities [diabetes, hypertension, chronic heart failure, chronic respiratory failure, chronic liver failure, chronic renal failure, immunodeficiency, recent or ongoing chemotherapy, chronic alcohol abuse, body mass index (BMI) > 30 kg/m^2^, malnourishment (BMI < 18.5 kg/m^2^ or weight loss > 10% over the previous 6 months)].
- Use of extracorporeal organ support such as extracorporeal membrane oxygenation and/or renal replacement therapy, use of catecholamines, mechanical ventilation (MV) settings, Glasgow score.
- Highest bilirubin level, lowest platelet level, lowest prothrombin level, and lowest PaO2/FiO2 ratio during ICU stay and PaO2/FiO2 ratio on the day of VAP onset.
- Simplified Acute Physiology Score II (SAPS II) within 48 h of admission and Sequential Organ Failure Assessment (SOFA) scores on admission and on the day of VAP onset.
- Data on infection: type of respiratory sample, type of infection (monomicrobial or polymicrobial), duration of previous MV, known rectal or respiratory colonization with *S. maltophilia*, and CPIS on the day of microbiological sampling.
- Susceptibility of *S. maltophilia* strains to selected antimicrobial agents, as determined using the disk-diffusion method and the minimum inhibitory concentrations defined by the European Committee on Antimicrobial Susceptibility Testing [15].
- Data on treatment of VAP caused by *S. maltophilia*: class of antibiotics, type of therapy (monotherapy or combination therapy), as well as time to start and duration of appropriate antibiotic therapy.
- Prognosis data: MV duration, length of stay in ICU and in hospital, ICU and hospital mortality.

### Statistical analyses

Patient characteristics were described as frequency and percentage for categorical variables and as median and interquartile range for quantitative variables. Qualitative variables were compared using the Chi-square test, the Kruskal–Wallis test or Fisher’s exact test, as appropriate. Continuous variables were compared using the nonparametric Mann–Whitney test. Bivariate analysis of the main outcome (ICU mortality) was performed using the Kaplan–Meier method and comparisons were performed using the log-rank test. A multivariate analysis was conducted with a Cox model. A competing risk analysis of the main outcome was also performed for patients who underwent Withholding of Life-Sustaining Treatment (WLST) versus those who did not. Adjusted hazard ratios (HR) and their 95% confidence intervals (CI) were calculated. The proportional risk hypothesis was tested by introducing an interaction between groups (treated patients versus non-treated patients) and time. All tests were performed at a 2-tailed type I error of 5% using SAS 9.4 software (SAS Institute Inc., Cary, NC).

## Results

### Population

Between January 2010 and December 2018, 9542 patients were hospitalized in our ICU, 130 (1.4%) of whom presented with VAP caused by *S. maltophilia.* Our study population was predominantly male (63.8%), with a median age of 61 [51–70] years. Median severity scores on admission were 9 [7–12] for SOFA and 58 [43–73] for SAPS II. Reasons for admission to ICU were acute respiratory failure in almost half of cases (48.5%), followed by septic shock (23.1%), post-operative management of cardiac surgery (21.5%), neurological causes, cardiogenic shock, and polytrauma. Lastly, 36.2% of our patients had chronic heart failure, 20.8% had chronic respiratory failure, 30.7% had chronic alcohol abuse, and 30.7% were malnourished (Table 1).

**Table 1:** Demographic and baseline characteristics of ICU patients with VAP caused by *S. maltophilia*

Variables	Total *N* = 130	Alive at ICU discharge *n* = 65	Dead at ICU discharge *n* = 65	*p*-value
Sex, male	83 (63.8)	41 (63.1)	42 (64.6)	0.85
Sex, female	47 (36.2)	24 (18.5)	23 (35.4)	
Age, years	61 [51–70]	56 [44–65]	63 [56–65]	< 0.001
Comorbidities				
Chronic respiratory failure	27 (20.8)	10 (15.4)	17 (26.2)	0.13
Chronic heart failure	47 (36.2)	18 (27.7)	29 (44.6)	0.04
Chronic renal failure	30 (23.1)	9 (13.8)	21 (32.3)	0.01
Immunodeficiency	6 (4.6)	2 (3.1)	4 (6.2)	0.68
Hypertension	64 (49.2)	27 (41.5)	37 (56.9)	0.08
Recent or ongoing chemotherapy	12 (9.2)	4 (6.1)	8 (12.3)	0.22
BMI > 30 kg/m^2^	29 (22.3)	14 (21.5)	15 (23.1)	0.83
Malnourishment	40 (30.7)	20 (30.8)	20 (30.8)	1
Diabetes	42 (32.3)	15 (23.1)	27 (41.5)	0.002
Severity scores				
SOFA score on admission	9 [7–12]	9 [7–11]	10 [7–12]	0.06
SAPS II	58 [43–73]	50 [37–68]	65 [50–76]	0.003
SOFA score on day of VAP onset	7 [4–11]	6 [3–9]	10 [6–12]	< 0.001

Quantitative variables are expressed as median [25–75th percentiles] and qualitative variables as number (%)

*ICU* intensive care unit, *VAP* ventilator-associated pneumonia, *BMI* body mass index, *SOFA* Sequential Organ Failure Assessment; *SAPS* Simplified Acute Physiology Score

### Sampling methods and microorganism distribution

Respiratory samples were obtained through endotracheal aspiration in 46.2% of cases, protected distal sampling in 34.6% of cases, and bronchoalveolar lavage in 19.2% of cases.

Infection was polymicrobial in 44.6% of cases. The microorganism most frequently associated with *S. maltophilia* was *Pseudomonas aeruginosa* (Table 3).

Median CPIS was 8 [7–9], and MV duration before onset of VAP was 12 [5–18] days (Table 2).

**Table 2:** Description and treatment of VAP caused by *S. maltophilia*

Variables	Total *N* = 130	Alive at ICU discharge *n* = 65	Dead at ICU discharge *n* = 65	*p*-value
Infection				
Bacteremia	4 (3.1)	1 (1.5)	3 (4.6)	0.89
Polymicrobial	58 (44.6)	32 (49.2)	26 (40)	0.29
Duration of mechanical ventilation before onset of VAP, days	12 [5–18]	13.5 [5.5–19]	10 [5–16]	0.62
Treatment				
No treatment	38 (29.2)	18 (13.8)	20 (15.4)	0.57
Monotherapy	4 (0.03)	3 (0.02)	1	
Combination therapy (≥ 2)	88 (67.7)	44 (67.7)	44 (67.7)	
Trimethoprim/sulfamethoxazole	80 (62.5)	40 (62.5)	40 (62.5)	1
Fluoroquinolone	84 (65.6)	40 (62.5)	44 (68.7)	0.46
Ticarcillin–clavulanate	39 (30.4)	20 (31.3)	19 (29.7)	0.85
Ceftazidime	9 (7.0)	5 (7.8)	4 (6.3)	0.99
Duration of appropriate antibiotic therapy, days	8 [5–14]	9[6–14]	7[3–12]	0.12
Time to start of appropriate antibiotic therapy, days	2 [1–3]	2 [1, 2]	2 [1–3]	0.58

Quantitative variables are expressed as median [25–75th percentiles] and qualitative variables as number (%)

*VAP* ventilator-associated pneumonia, *ICU* intensive care unit, *IQR* interquartile range

### Susceptibility of *Stenotrophomonas maltophilia* strains and type of antibiotic therapy

Identified strains of *S. maltophilia* were susceptible to trimethoprim–sulfamethoxazole in 86.2% of cases, to fluoroquinolones in 85.4% of cases, to ticarcillin–clavulanate in 58.5% of cases, and to ceftazidime in 40.0% of cases. Appropriate antibiotic therapy was initiated after a median of 2 [1–3] days.

Dual antibiotic therapy was initiated in 53 (40.8%) patients, 43 (81.1%) of whom received trimethoprim–sulfamethoxazole and fluoroquinolone. Triple antibiotic therapy was initiated in 35 (26.9%) patients, 32 (91.4%) of whom received ticarcillin–clavulanate, trimethoprim–sulfamethoxazole, and fluoroquinolone (Table 3). A total of 38 patients (29.2%) received no treatment for *S. maltophilia*. There was no significant difference between treated and untreated patients with respect to the CPIS (*p* = 0.17), the SOFA score on the day of VAP onset (*p* = 0.43), the polymicrobial character of the infection (*p* = 0.12), or the presence of WLST (*p* = 0.55). Probabilistic antibiotic therapy was effective against the co-infecting microorganism(s) in 86.2% of cases (50/58) and in 100% of cases when the co-infecting microorganism was *P. aeruginosa*.

**Table 3:** Microbiological description of ventilator-associated pneumonia caused by *S. maltophilia*

Variables	Total *N* = 130
Monomicrobial (*S. maltophilia*)	72 (55.4)
Polymicrobial	58 (44.6)
*Acinetobacter *spp.	4
*Citrobacter *spp.	1
*Enterobacter *spp.	10
*Enterococcus* spp.	5
*Escherichia coli*	2
*Klebsiella *spp.	5
*Proteus *spp.	4
*Pseudomonas aeruginosa*	24
*Serratia *spp.	3
*Staphylococcus *spp.	6
*Other*	1

Qualitative variables are expressed as number (%)

### Prognosis and risk factors associated with ICU mortality

Median MV duration was 21 [14–37] days and median MV duration after onset of VAP was 7 [4–15] days. Tracheotomy was performed in 31.5% of patients, with a statistically higher incidence in surviving patients [41.5% in survivors versus 21.5% in non-survivors (*p* = 0.01)]. ICU mortality was 50.0% and hospital mortality was 56.2%.

**Fig. 2:**
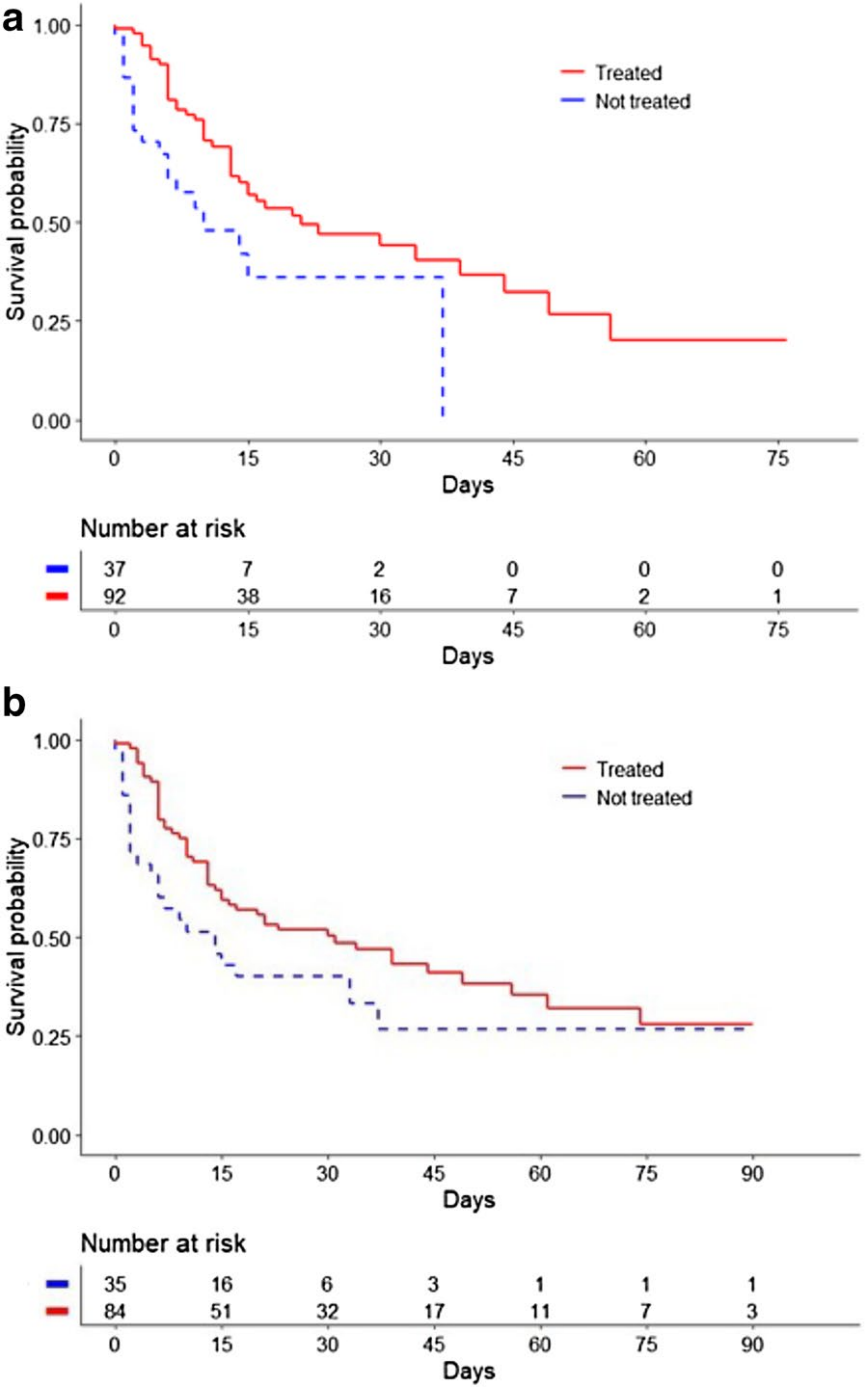
Kaplan–Meier estimates of the probability of survival. **a** ICU Survival according to an appropriate antibiotic treatment against *S. maltophilia.* **b** Hospital survival according to an appropriate antibiotic treatment against *S. maltophilia*

**Fig. 3:**
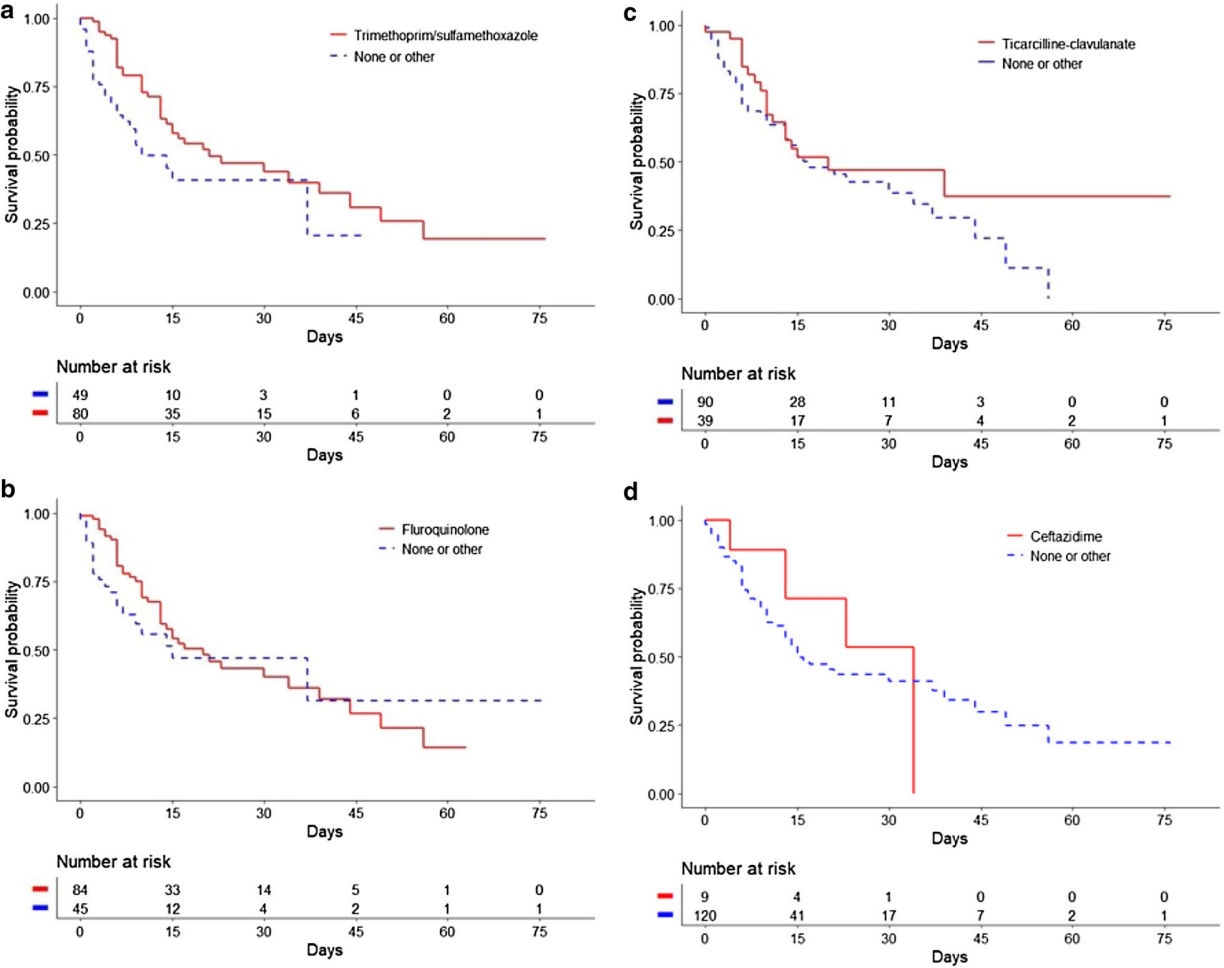
Kaplan–Meier estimates of the probability of ICU Survival. **a** ICU survival according to the antibiotic treatment with trimethoprim/sulfamethoxazole. **b** ICU survival according to antibiotic treatment with fluoroquinolone. **c** ICU survival according to antibiotic treatment with ticarcillin/clavulanate. **d** ICU survival according to antibiotic treatment ceftazidime

The Kaplan–Meier method using the log-rank test found a significant difference in ICU survival and hospital survival between treated and non-treated patients (*p* = 0.009 and *p* = 0.02, respectively) (Fig. 2). The only antibiotic therapy associated with a significant difference between treated and non-treated patients was trimethoprim–sulfamethoxazole (*p* = 0.02) (Fig. 3). For the others, there was no difference between treated and non-treated patients: fluoroquinolones (*p* = 0.54), ticarcillin–clavulanate (*p* = 0.14), and ceftazidime (*p* = 0.64).

After adjustment for confounding factors (Table 4), appropriate antibiotic therapy was associated with decreased ICU mortality (HR: 0.42; 95% CI 0.24–0.74, *p* = 0.003) and decreased hospital mortality (HR: 0.47; 95% CI 0.28–0.79, *p* = 0.04). When taking competing risks into consideration (Table 5), appropriate antibiotic therapy was associated with a similar significant decrease in ICU mortality in patients who underwent WLST (HR: 0.47; 95%CI 0.24–0.90; *p* = 0.02) and in patients who did not (HR: 0.33; 95% CI 0.11–0.97; *p* = 0.04).

**Table 4:** Risk factors independently associated with ICU mortality in the 130 patients assessed by multivariate Cox regression analysis

Variables	Hazard ratio (CI 95%)	*p*-value
Appropriate treatment	0.42 (0.24–0.74)	0.003
Age	1.03 (1.01–1.04)	0.003
SOFA score on day of VAP onset	1.07 (1.02–1.13)	0.006

Qualitative variables are expressed as number (%)

*ICU* intensive care unit, *CI* confidence interval, *SOFA* Sequential Organ Failure Assessment, *VAP* ventilator-associated pneumonia

**Table 5:** Competing risk factors independently associated with ICU mortality in the 130 patients assessed by multivariate Cox regression analysis

Variables	Hazard ratio (CI 95%)	*p*-value
Death without WLST		
Appropriate treatment	0.33 (0.11–0.97)	0.04
Age	1.02 (0.98–1.05)	0.35
SOFA score on day of VAP onset	1.12 (1.01–1.24)	0.04
Death with WLST		
Appropriate treatment	0.47 (0.24–0.90)	0.02
Age	1.03 (1.01–1.05)	0.004
SOFA score on day of VAP onset	1.12 (1.01–1.24)	0.05

Qualitative variables are expressed as number (%)

*CI* confidence interval, *WLST* Withholding of Life-Sustaining Treatment, *SOFA* Sequential Organ Failure Assessment, *VAP* ventilator-associated pneumonia

## Discussion

We performed a search of the literature in English and French using the terms pneumonia, *S. maltophilia*, intensive care unit, and outcome. We found three articles on VAP caused by *S. maltophilia*, two of which were conducted in adult populations. The first was the multicenter study by *Guerci *et al*.* (236 cases) and the second the study by *Ibn Saied *et al. (102 cases from the OUTCOMEREA database) [2, 16]. Both studies examined the impact of therapeutic modalities (antibiotic combination, treatment duration, etc.) on the prognosis of patients, but neither of them evaluated the association between prognosis and lack of appropriate treatment.

As in other studies of VAP caused by *S. maltophilia* [4, 16], the risk factors associated with increased ICU mortality were older age and high SOFA score on the day of VAP onset.

An original finding of our study was that appropriate antibiotic therapy*,* and in particular trimethoprim–sulfamethoxazole, is associated with decreased ICU mortality (HR: 0.42; 95% CI 0.24–0.74, *p* = 0.003) and hospital mortality (HR: 0.47; 95% CI 0.28–0.79, *p* = 0.04). However, time to start of appropriate antibiotic therapy and duration of appropriate antibiotic therapy had no effect on mortality.

Most studies of ICU patients with pneumonia caused by *S. maltophilia* found no association between mortality and antibiotic therapy strategy [2, 16]. The only exceptions are the study by *Tseng *et al*.* [17] and *Hanes *et al*.* [7], in which mortality was associated with delayed initiation of appropriate antibiotic therapy. We found no such association in our study, even though appropriate antibiotic therapy was initiated 2 days after VAP onset. The discrepancy between our results and those of *Hanes *et al. may be partly explained by the fact that the latter found a higher rate of co-infection in their cohort, and in particular a higher rate of co-infection with *P. aeruginosa* (92.3% of patients with a co-infection and 34.6% with a co-infection with *P. aeruginosa* versus 44.6% with a co-infection and 18.5% with a co-infection with *P. aeruginosa* in our study) [7]. Likewise, in the study by *Tseng *et al., the association between mortality and delayed initiation of appropriate antibiotic therapy was especially strong in cases of co-infection [17]. In line with these findings, *Yin *et al. observed that co-infection with *P. aeruginosa*, which is common in cases of *S. maltophilia* pneumonia, is a poor prognostic factor [18]. In our study, however, the rate of co-infection was the same in survivors and non-survivors (*p* = 0.29).

Studies examining VAPs caused by non-fermenting Gram-negative bacilli (GNB) found no effect of treatment duration on mortality, MV duration, or length of stay in ICU. However, in the randomized prospective study by *Chastre *et al., prolonged antibiotic therapy (15 days) was associated with fewer relapses than short antibiotic therapy (8 days) in the subgroup of patients infected with non-fermenting GNB (41.0% of relapses in patients who received 8 days of treatment versus 25.0% in patients who received 15 days of treatment) [19]. Similarly, in a systematic review of prolonged treatment for hospital-acquired pneumonia, *Pugh *et al. observed fewer relapses in patients infected with non-fermenting GNB who had received prolonged treatment (OR: 2.18; 95% CI 1.1–4.2) [20]. It should be noted, however, that the most frequently implicated non-fermenting GNB in the evaluated studies was *P. aeruginosa* [19, 20]. A randomized study is currently underway that evaluates the effect of treatment duration on the prognosis of patients with VAP caused by *P. aeruginosa* [21].

In our study, patients were treated mainly with combination therapy, with no difference in effect on mortality between dual and triple therapy. The difference in effect on mortality between combination therapy and monotherapy could not be evaluated, as only 4 of our patients received monotherapy. Our study suggests that trimethoprim–sulfamethoxazole has a protective effect in patients with VAP caused by *S. maltophilia*. This finding is in line with other studies of patients with *S. maltophilia* pneumonia, in which trimethoprim–sulfamethoxazole was not associated with excess mortality or the emergence of resistance, even when used as monotherapy [2, 22–24]. One in vitro study did find that combination therapy broadens the spectrum of antibiotics and constitutes as such a more effective probabilistic treatment for patients with sepsis, particularly in cases of non-fermenting GNB infection [25]. However, clinical studies found no superiority of combination therapy over monotherapy in cases of sepsis, whether in terms of clinical cure or mortality, and this even in cases of non-fermenting GNB infection [26, 27].

The incidence of VAP caused by *S. maltophilia* was relatively high (1.4%) in our ICU compared to what has been reported in the literature. *Ibn Saied *et al*.* and *Guerci *et al*.* reported an incidence of 0.5% and 0.3%, respectively—keeping in mind that the latter study examined all cases of hospital-acquired *S. maltophilia* pneumonia [2, 16]. The study by *Nseir *et al*.* found an incidence of 2.0%, but it included cases of colonization with *S. maltophilia* in addition to infection cases [3]. The discrepancy between these and our results may be explained by the fact that our study was conducted in a tropical environment, which has been shown to favor the development of the parent microorganism *Acinetobacter baumannii* [28]. Another potential explanation is that our patients were diagnosed using the highly sensitive sampling technique of endotracheal aspiration.

In our study, ICU mortality was 50.0% and hospital mortality was 56.2%. These findings are in line with the study by *Saugel *et al*.,* in which ICU mortality was 50.0% [4]. While *Guerci *et al. found a hospital mortality of 50.0%, their study included all cases of hospital-acquired pneumonia caused by *S. maltophilia* and not just those of VAP [2]. The study by *Ibn Saied *et al., in which the median SOFA score on the day of VAP onset was the same as in our study, found an ICU mortality of 40.0% despite the fact that 68.0% of patients received no treatment [16]. It may be that *Ibn Saied *et al. included cases of colonization with *S. maltophilia* in addition to cases of infection in their sample, which could explain this lower ICU mortality [16]. The study by *Saugel *et al*.*, in which ICU mortality was 29.0% in colonized patients, would tend to support this hypothesis [4].

Our study has some limitations. The single center and retrospective nature of the study may have led to biases. In addition, the relatively small size of our sample limits the statistical power of the results. The size of our sample stems from our decision to exclude patients with a CPIS ≤ 6 who were not treated by a clinician for VAP, as we assumed these to be cases of colonization with *S. maltophilia* or ventilator-associated tracheobronchitis [4, 29]. It should be noted, however, that the retrospective studies by *Guerci *et al*.* and *Ibn Saied *et al*.* included a comparable number of patients: 228 (80.0% of whom had VAP) and 102, respectively [2, 16].

Another limitation of our study is that we did not collect data on relapse or emergence of resistance. Lastly, we used mostly endotracheal aspiration with quantitative cultures, which may have led us to overestimate the incidence of *S. maltophilia* in our population due to heightened sensitivity. Current guidelines for the management of VAP allow the use of endotracheal aspirates (in addition to invasive respiratory specimens), but with semi-quantitative as opposed to quantitative cultures. In France, however, endotracheal aspiration with quantitative cultures is the most commonly used technique for the diagnosis of VAP [30]. Thus, in the study by *Guerci *et al., which represents the largest sample of patients with VAP caused by *S. maltophilia* to date, 30.0% of cases were diagnosed using this technique [2].

## Conclusion

This is the third retrospective study of VAP caused by *S. maltophilia* to use a large sample of patients and the first to focus primarily on the effect of antibiotic therapy on the prognosis of infected patients.

In our study, appropriate antibiotic therapy, and in particular trimethoprim–sulfamethoxazole, was associated with decreased ICU and hospital mortality in patients with VAP caused by *S. maltophilia*.

## Data Availability

The datasets used and/or analyzed for this study are available from the corresponding author on reasonable request.

## Abbreviations

VAP: Ventilator-associated pneumonia
ICU: Intensive care unit
CFU: Colony forming unit
CPIS: Clinical Pulmonary Infection Score
BMI: Body mass index
MV: Mechanical ventilation
SAPS II: Simplified Acute Physiology Score II
SOFA: Sequential Organ Failure Assessment
HZ: Hazard ratio
CI: Confidence interval
WLST: Withholding of Life-Sustaining Treatment
GNB: Gram-negative bacilli
